# Accurate Characterization of Bladder Cancer Cells with Intraoperative Flow Cytometry

**DOI:** 10.3390/cancers14215440

**Published:** 2022-11-04

**Authors:** Athanasios Paliouras, Georgios S. Markopoulos, Stavros Tsampalas, Stefania Mantziou, Ioannis Giannakis, Dimitrios Baltogiannis, Georgios K. Glantzounis, George A. Alexiou, Evangelia Lampri, Nikolaos Sofikitis, George Vartholomatos

**Affiliations:** 1 Department of Urology, University Hospital of Ioannina, 45500 Ioannina, Greece; 2 Haematology Laboratory, Unit of Molecular Biology and Translational Flow Cytometry, University Hospital of Ioannina, 45500 Ioannina, Greece; 3 Neurosurgical Institute, University of Ioannina Faculty of Medicine, 45500 Ioannina, Greece; 4 Hepato-Pancreatico-Biliary (HPB) Unit, Department of Surgery, University Hospital of Ioannina, and Faculty of Medicine, University of Ioannina, 45500 Ioannina, Greece; 5 Department of Neurosurgery, University Hospital of Ioannina, 45500 Ioannina, Greece; 6 Department of Pathology, University Hospital of Ioannina, 45500 Ioannina, Greece

**Keywords:** cancer, bladder cancer, flow cytometry, surgical treatment, surgical oncology

## Abstract

**Simple Summary:**

Bladder cancer is a malignancy that predominantly affects male patients. Surgical treatment is the first option for clinical management and cancer cell characterization is critical for tumor margin detection and complete tumor removal. We developed a specialized intraoperative flow cytometry (iFC) methodology for bladder cancer cell detection. Our study, including 52 individuals, reveals that iFC is highly specific, sensitive and accurate in detecting cancer cells, based on the quantification of cell proliferation and the presence of tumor aneuploidy. The results of this study advocate further research on the utility of iFC as a next-generation malignancy evaluation technique during transurethral resections.

**Abstract:**

Bladder cancer represents a major health issue. Transurethral resection is the first line treatment and an accurate assessment of tumor margins might warrant complete tumor removal. Genomic instability and proliferative potential are common hallmarks of cancer cells. We have previously demonstrated the utility of intraoperative flow cytometry (iFC), a next-generation margin evaluation methodology for assessment of DNA content, in the detection of several types of malignancy. In the current study we investigated the possible value of iFC in the characterization of bladder cancer during surgery. Samples from a population of 52 people with urothelial cancer were included in the study. The total time for iFC evaluation is 3–5 min per sample and included a two-step analysis, including DNA-index and Tumor-index calculation. First, DNA-index calculation revealed 24 hyperploid and one hypoploid tumor. Second, cell cycle analysis and Tumor-index calculation revealed that tumor samples are distinguished from normal cells based on their significantly higher proliferative potential. The standard for iFC evaluation was pathology assessment and revealed that our protocol exhibits an accuracy of 98% in defining the presence of cancer cells in a given sample. Our results support the further assessment of iFC value towards its use as a novel malignancy evaluation tool in transurethral resections.

## 1. Introduction

Cancer remains a leading cause of human mortality with a worldwide rate of more than 1 death per 1000 people per year [1]. Amongst urological cancers, bladder cancer is the most common in the male urinary tract, displaying two to six times elevated frequency in male than female patients [2] and recording 440,864 cases and 158,785 deaths in male patients during 2020, according to Global Cancer Observatory [1]. Approximately 90–95% of bladder cancer cases are attributed to transformation of urothelial cells, lining throughout the urinary tract, in to cancer cells leading to urothelial carcinomas whereas squamous cell carcinoma, originating from bladder-lining cells, as well as adenocarcinoma [3] deriving from glandular cells account for ~4 and 2% of cases, respectively.

Depending on the depth of invasion, bladder cancer incidents are classified either as non–muscle-invasive (NMIBC) which are characterized by lower metastatic potential and more favorable prognosis (80% of total cases) or as muscle-invasive bladder cancer cells (MIBC) that reflect increased invasiveness and progress to metastatic disease [4]. ΜΙΒC cases may arise by remission of NMIBC cases but the majority of them are de novo diagnoses with a 5-year survival rate less than 60% at local disease and less than 10% in distant metastases [4,5]. Due to distinct histopathological features and heterogeneity deriving from differentially expressed genes [6,7], NMIBC and MIBC can be further divided into the subtypes of basal- and luminal-like and subdivided by TCGA (The Cancer Genome Atlas) into luminal-papillary, infiltrated, squamous, neuronal/small cell and luminal/genomically unstable (GU) carcinomas [8]. Recently, luminal subtype was further subcategorized to a basal-squamous and distinct neuronal carcinoma (SCC) [6]. Genomic instability is a recognized hallmark of bladder cancer, leading on defects on cell cycle and growth regulation [9] thus giving rise to malignant transformation and sustaining proliferation [10].

Immunohistological classification and the molecular characteristics in bladder carcinomas are a cornerstone in evaluating and addressing subsequent treatment strategy as distinct cells may present differential response to anti-cancer drugs [11,12,13]. Whilst bladder cancer is detected predominantly in early stages (7 in 10 cases), thus being candidate for tumor resection [14], unlikely 15–70% of cases relapse after one year. Following successful removal, the 5-year survival rate is 94% for in situ tumors and falls to 6% in metastatic cases [15,16].

Surgical management is the first-line treatment for 4 out of 5 cancer cases, since it has been estimated that more than 80% of the >15 million cancer cases in 2015 were candidates for surgical treatment [17]. The low mortality rate and morbidity following surgery and the prospective of complete tumor removal are among the main advantages, making surgery a method of choice, with a projection that by 2030, >45 million surgical procedures will be performed regarding tumor removal [17]. Transurethral resection is the typical methodology for both diagnosis and therapy of non-invasive bladder cancer [18]. The success of the operation is based on several variables and requires the accurate characterization of cancer cells for both diagnosis and the further therapeutic management of patients [18].

Flow Cytometry is among the most effective single-cell analysis technologies with several applications in cancer: immunophenotyping, characterization of hematological malignancies, revealing measurable residual disease, ploidy and cell cycle assessment, among others [19]. Intraoperative flow cytometry (iFC) is a rapid, highly sensitive and relatively inexpensive method with the potential of characterizing tumor biology and margin status, offering a potential of complete tumor removal. Our team has utilized intraoperative flow cytometry, originally for brain malignancies [20,21,22,23]. Based on the high diagnostic potential of iFC, the methodology has further been standardized and applied in several types of malignancy, including head and neck [24,25], breast [26,27,28], liver [29], pancreatic [30] colorectal [31], as well as gynecological neoplasms [32]. Since iFC facilitates near real time detection of aneuploidy and calculation of S- plus G2/M-phase fraction in surgical samples, it offers an accurate measurement of cancer hallmarks in 3–5 min. In conclusion, iFC is a powerful informative tool towards discrimination of tumor cells from surrounding healthy tissue in a plethora of malignancies leading to optimal resection and remission prognosis. The current study is, to our knowledge, the first application of iFC in bladder cancer and urological malignancies. We optimized previous iFC protocols for ideal performance in sample collection and analysis in transurethral resections. Our data suggest that iFC provides a rapid and accurate (98.1%) cancer characterization and warrants further examination in larger studies.

## 2. Materials and Methods

### 2.1. Study Sample

The study included patients that underwent transurethral resection between 2021–2022, in the Department of Urology, University Hospital of Ioannina (UHI). All operations were performed by urologists. The total number of patients was 52, which were enrolled following an informed consent. The study had approval of the Institutional Review Board of UHI and was in accordance with the principles of the Declaration of Helsinki.

During surgery, a sample (~5 mm^2^ of volume) was taken from the tumor tissue, while an additional sample from macroscopically healthy tissue. Both samples were blinded, divided into equal pieces used for flow cytometry analysis and pathology evaluation, respectively. Pathology assessment, performed by an expert pathologist, was considered as the standard for evaluation.

### 2.2. DNA Content Analysis

DNA analysis was performed following tumor excision according to Ioannina Protocol, a methodology for intraoperative assessment first implemented for brain malignancies [21]. In short, samples were minced through a Medimachine (BD Bioscience) for 1 min in standard phosphate buffered saline buffer and a cell suspension was obtained. Suspended cells were diluted to a final concentration of 10^6^ cells/mL, following an automated hematology analyzer count. The final homogenized cell suspension was stained, using a propidium iodide solution (125 μg/mL) for 3 min and immediately processed for flow cytometric analysis. For iFC analysis, a FACSCalibur flow cytometer was used, utilizing CellQuest software V3.1 (Both by BD Bioscience). A ficoll gradient on peripheral blood from healthy donors (Ficoll-Paque separation, GE Healthcare), was utilized to obtain peripheral blood mononuclear cells (PBMCs). PBMCs were designated as the normal standard for detecting the diploid peak of cells in G0/G1 phase. A typical number of 5000 gated events (stained cell nuclei) per sample were evaluated.

A post-acquisition analysis was performed to calculate DNA-index and Tumor-index. DNA-index represent the ploidy status of each cell and is calculated as a ratio between the geometric mean corresponding to G0/G1 peak of the sample to that of PBMCs. Hence, a DNA-index > 1.1 represents a hyperploid cell, while a DNA-index < 0.9 is indicative of a hypoploid cell, while DNA-index = 1, corresponds to diploid cancer. The Tumor-index specifies the cancer cell proliferation rate and is calculated as a cumulative percentage of cells in both S and G2/M phase.

### 2.3. Histopathological Assessment (Haematoxylin and Eosin Staining)

Surgical specimens of bladder tissues were paraffin-embedded for haematoxylin and eosin (H&E) staining and histopathological assessment. Dako Coverstainer (Agilent Technologies) was used for H&E process from baking, dewaxing and staining through to the dehydrated, coverslipped and dried slide, according to manufacturer’s instructions. The tissue sections were examined under a light microscope (Olympus BX41).

### 2.4. Statistical Analysis

Independent Samples Mann-Whitney U test was utilized to delineate whether the G0/G1 and Tumor-index between cancer and normal cells is significantly different. Receiver-Operating Characteristic (ROC) analysis has been utilized calculate the sensitivity and specificity of the assay and to determine the optimal cut-off value. Based on the ROC analysis results, each cut-off value was assessed to define the optimal accuracy of our assay. In each test, the level of significance was defined based on a probability (*p*-value) < 0.05. Data analysis was performed using SPSS software, version 23 (IBM) and further represented in Graphpad Prism, version 8.4.2 (Graphpad Sotware, LLC, San Diego, CA, USA).

## 3. Results

### 3.1. Study Population

The purpose of the current study was to assess the utility of intraoperative flow cytometry during transurethral resection. To this end, we studied a population that included individuals with bladder cancer that underwent a transurethral resection as a first line of treatment. In general, 52 patients were eligible for inclusion, with the vast majority being males (45 out of 52). Among the analysed tumors, 20 were low-grade and 32 were high-grade, according to pathology assessment. As regards tumor type, we included 41 cases of papillary urothelial carcinoma (PUC), 10 of Invasive urothelial carcinoma (IUC) and one case of squamous cell carcinoma of the urinary (SCCUB), respectively. Patients’ characteristics regarding tumor grade and type are shown in Table 1.

### 3.2. Intraoperative Flow Cytometry Determines the Presence of Bladder Cancer Cells with a High Sensitivity and Specificity 

Next, we performed iFC analysis in 52 individuals undergone urothelial resection and calculated for each sample the DNA-index and Tumor-index, two indices that support intraoperative cancer cell detection. In each case, the presence of a tumor has been identified and verified by pathological assessment.

**Figure 1:**
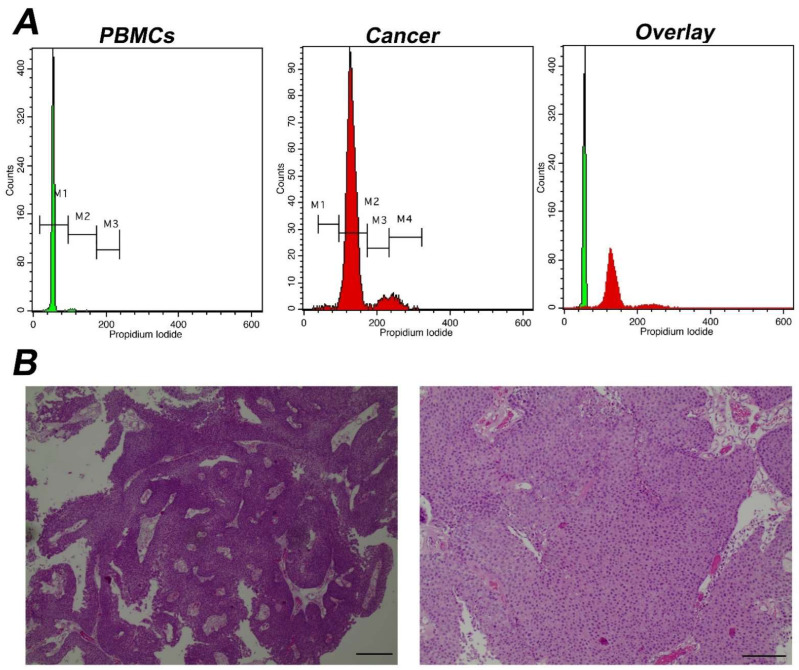
Analysis of a representative aneuploid tumor with high DNA-index (**A**) Analysis using iFC of DNA content and distribution of cell cycle phases. Peripheral blood mononuclear cells/PBMCs (**left panel**) and tumor cells obtained during surgery (**middle panel**) were stained with propidium iodide, to bind nucleic acids. The presented histograms are separated using 3 different marked areas, by respective markers (M1, M2 and M3), in the control PBMC cells. The areas correspond to cells that are in the G1, S, G2/M phases, respectively, based on the mean fluorescence. Cells in G2/M emit a dual mean fluorescence intensity than that of cells in G1, while cells in S phase (DNA replication phase) are characterized by an intermediate fluorescence intensity. In the tumor sample, the G0/G1 peak was coincided with M2 marker due to aneuploidy. Due to that, an additional marker, M4 was introduced and in this case, markers M2, M3 and M4 represent G1, S, G2/M phases, respectively. The DNA-index is 1.7 (**B**) pathology assessment. A papillary urothelial carcinoma of the bladder, low-grade, following hematoxylin-eosin staining. Original magnification ×40 (**left panel**) and ×100 (**right panel**). Scale bars represent 50 um (**left photo**) and 100 um (**right photo**), respectively.

**Figure 2:**
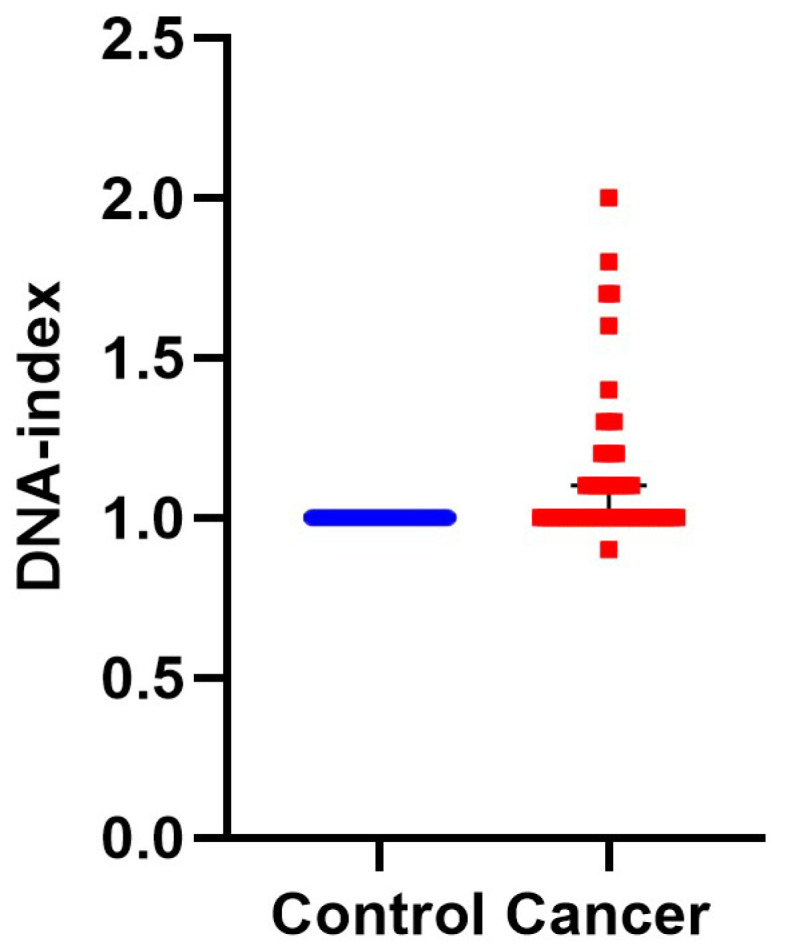
DNA-index represents aneuploidy of tumor cells. DNA-index values have been quantified by iFC and plotted, representing normal (blue dots) and cancer (red dots) cells in individual patient samples, respectively. While normal cells (taken from normal bladder tissue) are diploid, a DNA-index of ≠1 is a common, unique hallmark of cancer cells. The Median DNA index is depictred as a black horizontal line in each group.

First, we calculated DNA-index, an indicator of aneuploidy, since it is associated with malignancy and the hallmark of genomic instability [33]. In 28 cases the samples were diploid and the rest aneuploid. Aneuploid samples were furher divided into: 24 hyperploid (DNA-index ranging from 1.1–2) and one hypoploid (DNA-index = 0.9). A characteristic case of hyperploidy with a DNA-index = 1.7 is presented in Figure 1, while the cumulative results are presented in Figure 2 and Table S1. In conclusion, iFC may accurately assess aneuploidy in tumor samples.

**Figure 3:**
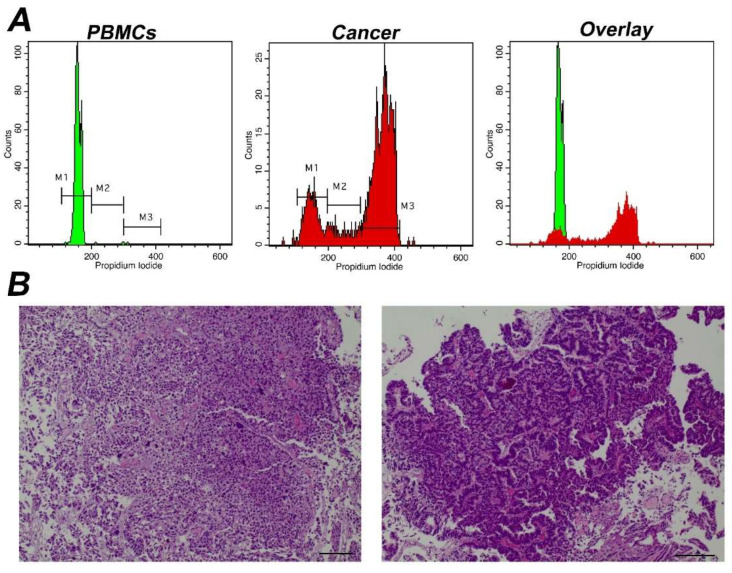
Analysis of a representative tumor with α high Tumor-index (**A**) Analysis using iFC of cell cycle distribution. Peripheral blood mononuclear cells/PBMCs (**left panel**) and tumor cells obtained during surgery (**middle panel**) were stained with propidium iodide, to bind nucleic acids. Markers M1, M2, M3 are used to gate cells in G1, S and G2/M phases, respectively, as explained in Figure 1. The cell fraction in each phase (presented in upper right corner of each histogram) is used to quantify Tumor-index, a marker used to determine the presence of malignant cells in a given sample. (**B**) pathology assessment. A papillary urothelial carcinoma of the bladder, high-grade, following hematoxylin-eosin staining. Original magnification ×40 (**left panel**) and ×100 (**right panel**). Scale bars represent 50 um (**left photo**) and 100 um (**right photo**), respectively.

Subsequently, in order to define cancer cell proliferative potential we performed a calculation of the percentage of G0/G1 cell cycle fraction and of Tumor-index (the cumulative cell population in both S and G2/M cell cycle phases) in each sample. Next, this calculation was utilized to predict the sensitivity and specificity of our study to distinguish cancer from normal cells. A typical analysis of a cancer sample with a high Tumor-index is depicted in Figure 3.

**Figure 4:**
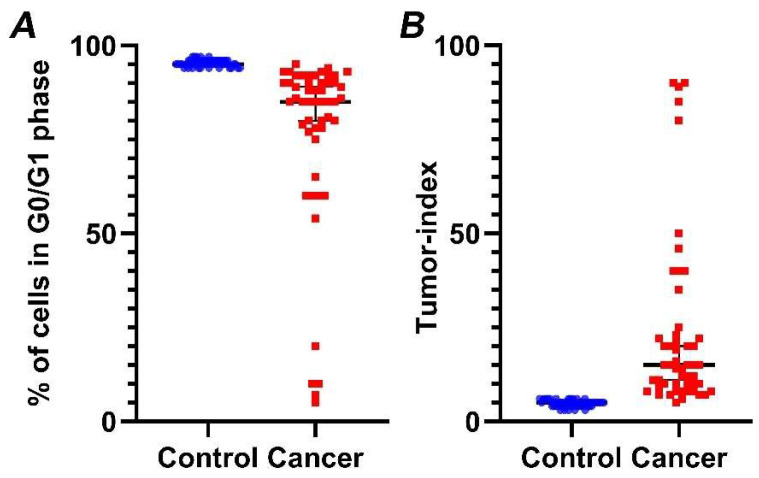
Percentage of cells in G0/G1 phase (**A**) and Tumor-index (**B**) in normal versus cancer samples, based on iFC. normal and cancer samples are presented as blue or red dots, respectively. Black horizontal lines represent median in each group. (**A**) Tumor samples exhibiting a significantly lower percentage in G0/G1 than normal bladder tissue. (**B**) Inversely, a significantly higher Tumor-index (the cumulative cell fraction in S and G2/M phases) is a hallmark of cancers cells.

Our analysis reveals that G0/G1 fraction in cancer cases is significantly lower (*p* < 0.01) (Figure 4A), while, in contrast, Tumor-index is significantly higher (*p* < 0.01) (Figure 4B), in relation to normal samples. Specifically, mean Tumor-index in normal tissue is 4.47 ± 0.12 (mean value ± standard error), while percentages of Tumor-index in tumor samples is 23.46 ± 3.25, respectively.

The results of the current study provide novel insights into intraoperative tumor analysis.

**Figure 5:**
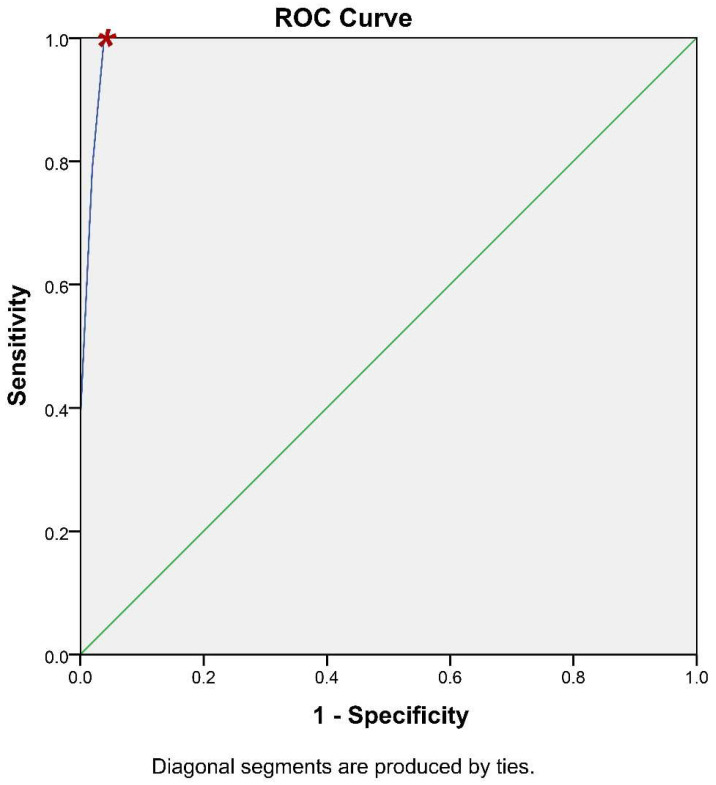
ROC-Curve analysis. iFC evaluation of neoplasia based on G0/G1 and Tumor-index calculation is highly accurate. Sensitivity (*Y*-axis) and 1- specificity (*X*-axis) for individual cut-off values is presented by the blue line. The selection of a spot in the blue line allows the calculation of the respective sensitivity and specificity, based on a cut-off value. The selected cut-off value is denoted in the curve with a red asterisk.

It is of paramount importance for a methodology of potential clinical significance to calculate its accuracy. To this end, the receiver operating characteristic (ROC) curve analysis has been utilized to assess whether iFC has the ability to accurately discriminate cancer tissue (Figure 5). Briefly, ROC analysis allows the plot between the sensitivity and specificity of an assay, based on different cut-off values. The calculation of the optimal value can be assisted by the accompanying table (Table S2), that allows the assessment of different cut-off values to define the optimal one. Based on ROC-analysis, the optimum cut-off value to delineate cancer is 93.5% in G0/G1 cell cycle fraction (or, inversely 6.5% in Tumor-index), which results in 100% sensitivity and 96.2% specificity (Table S2). The Positive Predictive Value is 96.2% and the Negative Predictive Value is 100%. Consequently, the accuracy of our assay is 98.1%. In conclusion, the iFC protocol we followed during transurethral resection is an accurate methodology to delineate cancer cells in a given sample.

**Figure 6:**
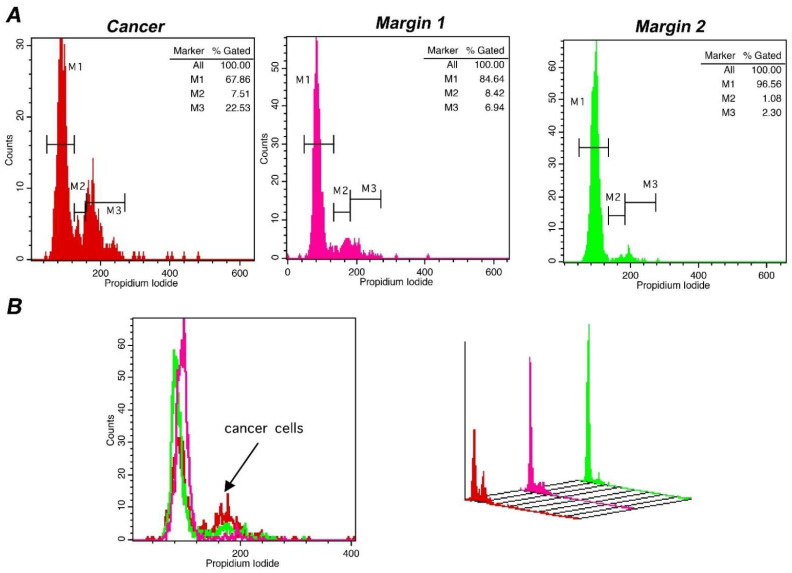
Margin assessment of a representative case of bladder cancer with iFC. DNA analysis of cancer cells, as described leads to quantification of cell cycle phases G0/G1. S and G2/M by the respective markers M1, M2 and M3. (**A**) DNA analysis of a tumor sample (red), a positive (pink) and a negative margin (green). Proliferating cancer cells (cumulative percentage in M2 and M3, or tumor-index) allows the assessment of margin status, where a percentage beyond the cut-off value of 6.5% is indicative of the presence of cancer cells in a given margin. In the presented case, margin 1 is positive, with a Tumor-index of ~15%, while margin 2 is negative, with a Tumor-index of ~3.4%, below the cut-off value. (**B**) overlays in 2 dimensions (**left**) and 3 dimensions (**right**). The presence of proliferating cancer cells is defined by the black arrow in the 2-D plot.

Lastly, in order to confirm the sensitivity of our methodology in defining tumor margins, we evaluated the status of tumor margins taken from different sites during surgery, in a subpopulation of 4 representative cases. In all cases, the margin status has been defined. Pathological assessment corroborated flow cytometry results with absolute agreement. The results of a representative case are presented in Figure 6, where also margin status analysis is described. In the presented case, DNA-index was 1 and the differences in the tumor-index was utilized to delineate margin status. The cut-off value of 6.5% in Tumor-index characterized margin 1 as positive and margin 2 as negative. Thus, margins containing different percentage of cancer cells in the total analyzed population exhibit distinct proliferation capacity, making it possible to delineate margin status. In conclusion, the accuracy of iFC in detecting cancer cells has been verified in selected cases with an absolute agreement to pathological assessment.

## 4. Discussion

Flow cytometry is inarguably the method of choice for measuring DNA content at the cellular level [34]. Among the main advantages of FC is the speed of analysis, accuracy, cost effectiveness and the fact that it can be applied in most tissues and cell lines derived from several cancer types [34], including bladder cancer [35]. The applications of FC in cancer include, among others, drug-screening and development on novel therapeutics [36,37,38], as well as diagnostic intraoperative FC analysis [22]. The intraoperative use of FC is contributing towards the accurate characterization of tumor biology and the resection margin status assessment. Our research team is working on the way to establish iFC as a universal next generation margin evaluation tool [28]. Till now, iFC has been successfully implemented in surgical procedures regarding malignancies of brain, head-and-neck, breast, gynecological, hepatobilliary and colorectum, with a high accuracy that in most cases is beyond 90% [20,21,22,23,24,25,26,27,28,29,30,31,32]. The current study extends the utility of iFC in bladder cancer analysis during transurethral resections and reveals that it exhibits a high accuracy (>98%) towards bladder cancer cell characterization. The assay exhibits a Negative Predictive Value of 100% and a Positive Predictive Value of 96.20%.

Although tumors that are diagnosed early can be managed by transurethral resection, the median survival following recurrence of invasive UC remains only 5.6 months. This notion underscores the importance of detection and therapeutic intervention of bladder cancer as early as possible [39,40]. The accurate tumor characterization is pivotal for post-operative management and to improve survival [41]. Based on DNA content analysis, we confirmed that iFC methodology can be expanded to assess and characterize malignancy during bladder cancer surgery. Our specialized methodology allowed for optimal sample collection and analysis. Consequently, along with correct identification of histological subtypes by pathology, evaluation of DNA-index and proliferation analysis of the resected tissue would be a strong ally in determining healthy tumor margins. Based on this notion, iFC may also assist in the prevention of muscle-invasive disease and spread of lymph node metastases [42] and, in all, improving oncological outcome. The utility of iFC in this type of surgery has been verified by a pilot analysis in selected samples, where margin status of five patients was assessed and margin status was successfully delineated, based on the golden standard pathology evaluation.

Genomic instability is an established hallmark of bladder cancer leading on defects on cell cycle and growth regulation [9] thus giving rise to malignant transformation and sustaining proliferation [10]. In fact, Ras-mitogen-activated protein kinase (MAPK) signal transduction pathway is altered in non-invasive carcinomas whilst two of the most frequently mutated tumor suppressor genes encountered in invasive tumors are retinoblastoma (RB) and p53 genes [43,44]. Luminal papillary tumor analysis revealed mutations in FGFR gene and aberrant FGFR3 and PIK3CA pathway activation [6,45]. Chromosomal aberrations (namely polyploidy and hypoploidy) are known causes of carcinogenesis, a hallmark of cancer cell cells as well as prognostic and predictive marker [34]. In our study, we detected aneuploidy in almost 50% of cancer cases, based on DNA-index calculation (Figure 2 and Table S1). In addition, the sustaining proliferative signaling of bladder cancer cells was detected as an induction of Tumor-index (Figure 4). We believe that the genomic instability, as a hallmark of bladder cancer cells, may be a reason behind the high sensitivity and specificity of iFC analysis (Figure 5).

## 5. Conclusions

Since surgical removal is the first line of treatment in most cancer cases, the ultimate goal in the field of surgical oncology is the complete tumor removal. A prerequisite for this, is the accurate characterization of a tumor sample, to delineate resection margins. In addition, the intraoperative representation of tumor biology would offer valuable information for further clinical management. Our report is the first to highlight the importance of iFC as a tool in transurethral resection that offers a rapid and accurate characterization of cancer cells, as well as a representation of tumor biology. These results need to be further evaluated and verified in larger populations and in multicenter studies.

## Figures and Tables

**Table 1:** Study population Characteristics.

Gender	
Male	45
Female	07
Grade ^1^	
Low	20
High	32
Type ^1,2^	
PUC	41
IUC	10
SCCUB	01

^1^ Based on Pathology Evaluation. ^2^ PUC: Papillary urothelial carcinoma. IUC: Invasive urothelial carcinoma. SCCUB: Squamous cell carcinoma of the urinary Bladder.

## Data Availability

The data presented in this study are available on request from the corresponding author.

## Supplementary Materials

The following supporting information can be downloaded at: https://www.mdpi.com/article/10.3390/cancers14215440/s1, Table S1: Descriptive statistics for DNA-index, percentage of cells in G0/G1 and Tumor-index in normal and cancer cells. Table S2: Roc curve analysis data. The cut-off value is designated with bold.

## Abbreviations

iFC: intraoperative flow cytometry; ROC curve: Receiver-Operating Characteristic curve; NMIBC: non–muscle-invasive bladder cancer; MIBC: muscle-invasive bladder cancer; H&E:haematoxylin and eosin staining; PUC: papillary urothelial carcinoma; IUC: Invasive urothelial carcinoma; SCCUB: squamous cell carcinoma of the urinary.
