# Supplementary material for: Accurate Characterization of Bladder Cancer Cells with Intraoperative Flow Cytometry
Source: Cancers (Basel). 2022 Nov 4;14(21):5440. doi: 10.3390/cancers14215440 (PMC9656620; doi:10.3390/cancers14215440)
Supplement: Supplementary file 1 [file cancers-14-05440-s001.zip › cancers-1998669-supplementary.pdf]

Table S1: Descriptive statistics for DNA-index, percentage of cells in G0/G1 and Tumor-index in normal and cancer cells.

Descriptives[a]

	Cancer = 1, Normal = 0			Statistic	Std. Error
DNA-Index	1,0	Mean		1,131	,0324
		95% Confidence Interval for Mean	Lower Bound	1,066	
			Upper Bound	1,196	
		5% Trimmed Mean		1,100	
		Median		1,000	
		Variance		,055	
		Std. Deviation		,2339	
		Minimum		,9	
		Maximum		2,0	
		Range		1,1	
		Interquartile Range		,2	
		Skewness		2,213	,330
		Kurtosis		4,540	,650
G0/G1	,0	Mean		95,269	,1262
		95% Confidence Interval for Mean	Lower Bound	95,016	
			Upper Bound	95,523	
		5% Trimmed Mean		95,244	
		Median		95,000	
		Variance		,828	
		Std. Deviation		,9100	
		Minimum		94,0	
		Maximum		97,0	
		Range		3,0	
		Interquartile Range		1,0	
		Skewness		,240	,330
		Kurtosis		-,682	,650
	1,0	Mean		76,558	3,3321
		95% Confidence Interval for Mean	Lower Bound	69,868	
			Upper Bound	83,247	
		5% Trimmed Mean		79,449	
		Median		85,000	
		Variance		577,350	
		Std. Deviation		24,0281	

		Minimum		5,0	
		Maximum		95,0	
		Range		90,0	
		Interquartile Range		12,8	
		Skewness		-2,094	,330
		Kurtosis		3,490	,650
Tumor-Index	,0	Mean		4,731	,1262
		95% Confidence Interval for Mean	Lower Bound	4,477	
			Upper Bound	4,984	
		5% Trimmed Mean		4,756	
		Median		5,000	
		Variance		,828	
		Std. Deviation		,9100	
		Minimum		3,0	
		Maximum		6,0	
		Range		3,0	
		Interquartile Range		1,0	
		Skewness		-,240	,330
		Kurtosis		-,682	,650
	1,0	Mean		23,462	3,2468
		95% Confidence Interval for Mean	Lower Bound	16,943	
			Upper Bound	29,980	
		5% Trimmed Mean		20,756	
		Median		15,000	
		Variance		548,175	
		Std. Deviation		23,4131	
		Minimum		5,0	
		Maximum		90,0	
		Range		85,0	
		Interquartile Range		13,5	
		Skewness		1,985	,330
		Kurtosis		3,011	,650

a. Dna-Index is constant (DNA index = 1) in normal cells. In this case, descriptive statistics analysis has been omitted.

Table S2: Roc curve analysis data. The cut-off value is designated with bold.

Coordinates of the Curve

Test Result Variable(s): G0/G1

Positive if Greater Than or Equal To[a]	Sensitivity	1 – Specificity
4,000	1,000	1,000
6,000	1,000	,981
8,500	1,000	,962
15,000	1,000	,923
37,000	1,000	,904
57,000	1,000	,885
62,500	1,000	,808
70,000	1,000	,788
76,000	1,000	,769
77,500	1,000	,750
78,500	1,000	,712
79,500	1,000	,692
80,500	1,000	,635
83,000	1,000	,615
85,500	1,000	,481
87,000	1,000	,442
88,500	1,000	,385
89,500	1,000	,346
91,000	1,000	,231
92,500	1,000	,115
**93,500**	**1,000**	**,038**
94,500	,788	,019
95,500	,385	,000
96,500	,096	,000
98,000	,000	,000

a. The smallest cut-off value is the minimum observed test value minus 1, and the largest cut-off value is the maximum observed test value plus 1. All the other cut-off values are the averages of two consecutive ordered observed test values.
